# Supplementary material for: The barley pan-genome reveals the hidden legacy of mutation breeding
Source: Nature. 2020 Nov 25;588(7837):284–9. doi: 10.1038/s41586-020-2947-8 (PMC7759462; doi:10.1038/s41586-020-2947-8)
Supplement: Supplementary file 1 — Supplementary Figure 1 | PCR-based genotyping of the 7H inversion. This is the original gel image from which the blue sections were cropped and used for Fig. 3. Morex and RGT planet were used as controls. All Valticky lines carry Morex allele of 7H inversion. Two Diamant lines (HOR 14972, HOR 4092) carry the RGT Planet allele, one Diamant line (HOR 2073) carries the Morex allele. In another two Diamant lines, neither the RGT Planet allele nor Morex allele was amplified. One cropped section in Fig. 3 does not contain molecular weight marker, but from the original image, it is clear that all correspond to correct fragment sizes. [file 41586_2020_2947_MOESM1_ESM.pdf]

nature research
https://doi.org/10.1038/s41586-020-2947-8

Supplementary information

The barley pan-genome reveals the hidden legacy of mutation breeding

In the format provided by the authors and unedited

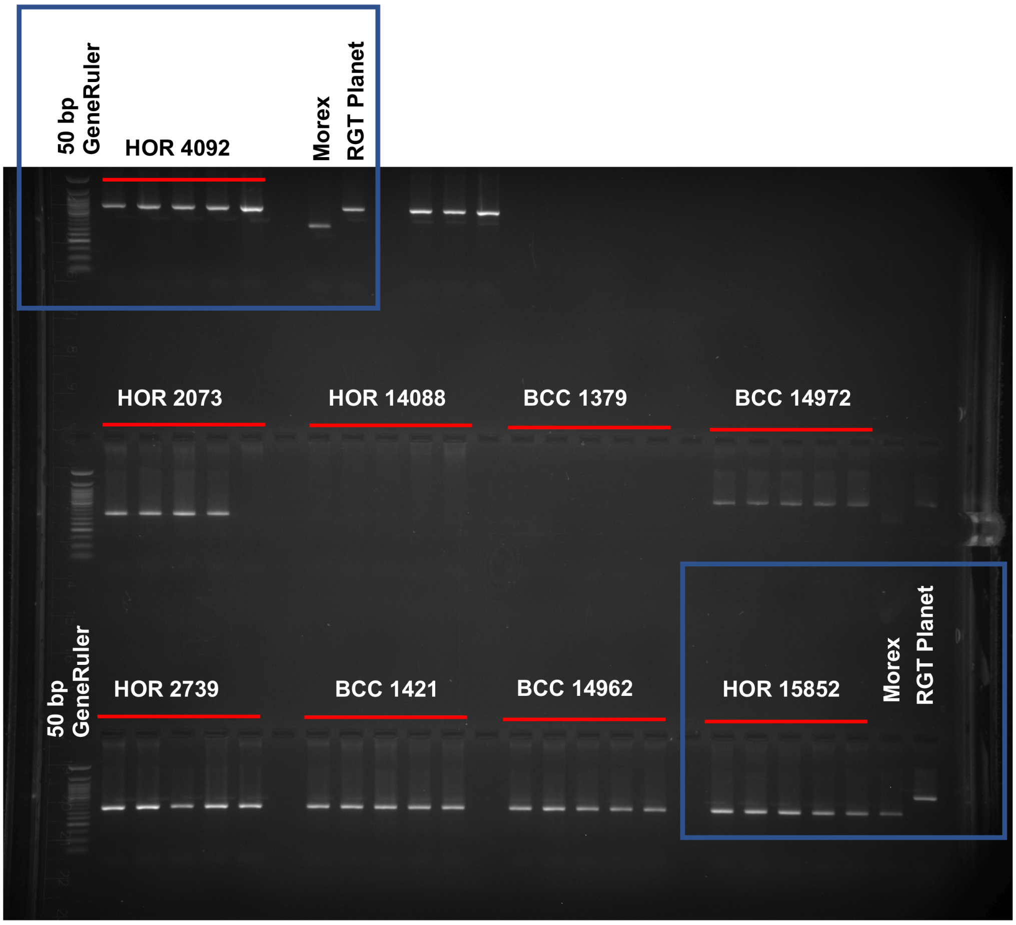

Accession	Name	7H allele
HOR 2739	Valticky	Morex/No inversion
BCC 1421	Valticky	Morex/No inversion
HOR 14962	VALTICKY	Morex/No inversion
HOR 15852	VALTICKY	Morex/No inversion
HOR 2073	Wadsacks Diamant	Morex/No inversion (4) + Another allele (1)
HOR 14088	SVALOEFS DIAMANT	Another allele (5)
BCC 1379	Diamant	Another allele (5)
HOR 14972	DIAMANT	RGT Planet/Inverted
HOR 4092	Diamant	RGT Planet/Inverted

Supplementary Figure 1 | PCR-based genotyping of the 7H inversion. This is the original gel image from which the blue sections were cropped and used for **Fig. 3**. Morex and RGT planet were used as controls. All Valticky lines carry Morex allele of 7H inversion. Two Diamant lines (HOR 14972, HOR 4092) carry the RGT Planet allele, one Diamant line (HOR 2073) carries the Morex allele. In another two Diamant lines, neither the RGT Planet allele nor Morex allele was amplified. One cropped section in **Fig. 3** does not contain molecular weight marker, but from the original image, it is clear that all correspond to correct fragment sizes.
