# Supplementary material for: The barley pan-genome reveals the hidden legacy of mutation breeding
Source: Nature. 2020 Nov 25;588(7837):284–9. doi: 10.1038/s41586-020-2947-8 (PMC7759462; doi:10.1038/s41586-020-2947-8)
Supplement: Supplementary file 2 — Reporting Summary [file 41586_2020_2947_MOESM2_ESM.pdf]

nature research

Corresponding author(s): Nils Stein, Martin Mascher, Chengdao Li

Last updated by author(s): Aug 25 2020

Reporting Summary

Nature Research wishes to improve the reproducibility of the work that we publish. This form provides structure for consistency and transparency in reporting. For further information on Nature Research policies, see Authors & Referees and the Editorial Policy Checklist .

Statistics

For all statistical analyses, confirm that the following items are present in the figure legend, table legend, main text, or Methods section.

n/a	Confirmed	
☐	☒	The exact sample size (*n*) for each experimental group/condition, given as a discrete number and unit of measurement
☒	☐	A statement on whether measurements were taken from distinct samples or whether the same sample was measured repeatedly
☐	☒	The statistical test(s) used AND whether they are one- or two-sided<br>*Only common tests should be described solely by name; describe more complex techniques in the Methods section.*
☒	☐	A description of all covariates tested
☒	☐	A description of any assumptions or corrections, such as tests of normality and adjustment for multiple comparisons
☒	☐	A full description of the statistical parameters including central tendency (e.g. means) or other basic estimates (e.g. regression coefficient) AND variation (e.g. standard deviation) or associated estimates of uncertainty (e.g. confidence intervals)
☐	☒	For null hypothesis testing, the test statistic (e.g. *F*, *t*, *r*) with confidence intervals, effect sizes, degrees of freedom and *P* value noted<br>*Give P values as exact values whenever suitable.*
☒	☐	For Bayesian analysis, information on the choice of priors and Markov chain Monte Carlo settings
☒	☐	For hierarchical and complex designs, identification of the appropriate level for tests and full reporting of outcomes
☒	☐	Estimates of effect sizes (e.g. Cohen's *d*, Pearson's *r*), indicating how they were calculated

Our web collection on statistics for biologists contains articles on many of the points above.

Software and code

Policy information about availability of computer code

Data collection	No software was used for data collection.
Data analysis	TRITEX assembly pipeline (commit: 7041ff2), NRGene DeNovoMagic v3.0, Minimap2 (version 2.17), Pipmaker (release 2011-08-12-01), GMAP (release 2018-07-04), SAMtools (v1.8), Novosort (V3.06.05), BCFtools (v1.8), R (v3.5.1), Assemblytics (v1.2.1), Isoseq pipeline (SMRTLink v5.0 Isoseq v1.0), blat (v35x1), exonerate (v2.2.0), AHRD (v3.3.3), OrthoFinder (v2.3.1), vmatch (2.3.0), genometools (1.5.9), BBDuk (BBMap_37.93), GAPIT (v3), igraph (v1.1.2), SNPRelate (v1.10.2), MapQTL (v6), MECAT (v1.1), Arrow (v2.3.3)

For manuscripts utilizing custom algorithms or software that are central to the research but not yet described in published literature, software must be made available to editors/reviewers. We strongly encourage code deposition in a community repository (e.g. GitHub). See the Nature Research guidelines for submitting code & software for further information.

Data

Policy information about availability of data

All manuscripts must include a data availability statement. This statement should provide the following information, where applicable:
- Accession codes, unique identifiers, or web links for publicly available datasets
- A list of figures that have associated raw data
- A description of any restrictions on data availability

All raw sequence data collected in this study and sequence assemblies have been deposited at the European Nucleotide Archive (ENA). Accession codes for raw data and assemblies are listed in supplementary tables: Supplementary Table 14 (assemblies), Supplementary Table 10 (assembly raw data), Supplementary Table 4 (WGS sequencing), Supplementary Table 5 (Hi-C), Supplementary Table 9 (DArT-seq). Assemblies, annotations and analysis results were deposited under a digital object identifier (DOI) in the PGP repository67 using the e!DAL submission system68 and accessible under the URL http://dx.doi.org/10.5447/ipk/2020/24. Assemblies and gene annotations can also be downloaded from https://barley-pangenome.ipk-gatersleben.de. The Barley Pedigree Catalogue is available at http://genbank.vurv.cz/barley/pedigree/.

Field-specific reporting

Please select the one below that is the best fit for your research. If you are not sure, read the appropriate sections before making your selection.

☒ Life sciences ☐ Behavioural & social sciences ☐ Ecological, evolutionary & environmental sciences

For a reference copy of the document with all sections, see nature.com/documents/nr-reporting-summary-flat.pdf

Life sciences study design

All studies must disclose on these points even when the disclosure is negative.

Sample size	Accessions for genome assembly were chosen to cover the diveristy (PCA) space of ~20,000 domesticated barley genotypes (Milner et al., 2019 Nature Genetics).<br>Accessions for resequencing (WGS, Hi-C) were chosen based on the core sets defined by Milner et al.<br>No sample size calculation was performed. Samples were chosen from major germplasm groups evident in PCA or because of relevance for barley genetics (Barke, Golden Promise, Igri)
Data exclusions	No data were excluded.
Replication	A partially replicated design was employed in field trials. No replication was done in genome assembly and sequencing.
Randomization	Field trials were done in randomized block designs. Experimental observations were done without predefined groupings. Other forms of randomization were not relevant to this study.
Blinding	Blind testing was not done as it was not relevant to plant molecular genetics and genomics work.

Reporting for specific materials, systems and methods

We require information from authors about some types of materials, experimental systems and methods used in many studies. Here, indicate whether each material, system or method listed is relevant to your study. If you are not sure if a list item applies to your research, read the appropriate section before selecting a response.

Materials & experimental systems

n/a	Involved in the study	
☒	☐	Antibodies
☒	☐	Eukaryotic cell lines
☒	☐	Palaeontology
☒	☐	Animals and other organisms
☒	☐	Human research participants
☒	☐	Clinical data

Methods

n/a	Involved in the study	
☒	☐	ChIP-seq
☒	☐	Flow cytometry
☒	☐	MRI-based neuroimaging
