# Supplementary material for: The barley pan-genome reveals the hidden legacy of mutation breeding
Source: Nature. 2020 Nov 25;588(7837):284–9. doi: 10.1038/s41586-020-2947-8 (PMC7759462; doi:10.1038/s41586-020-2947-8)
Supplement: Supplementary file 3 — | In-depth analysis of two inversions on 2H and 7H. (a) Schematic illustration showing precise positions of breakpoints for 7H frequent inversion between Morex and RGT Planet. (b) PCR assay for genotyping 7H inversion. The location of three PCR primers are shown in (a) with yellow marks (not drawn to scale). (c) PCR assay for genotyping the 2H inversion. Primer locations are shown in Fig. 4c. (d) Hi-C contact probability matrix of RGT Planet computed for chromosome 7H. The intensity of pixels represents the normalized Hi-C links counted between 1 Mb windows on chromosome 7H. The frequent 7H inversion was spotted as a pattern of higher than expected interaction frequency against Morex V2 reference genome, marked by blue lines. (e) QTL results for grain yield, plant height and different growth stages from multiple sites in RGT Planet x Hindmarsh population. [file 41586_2020_2947_MOESM3_ESM.pdf]

a

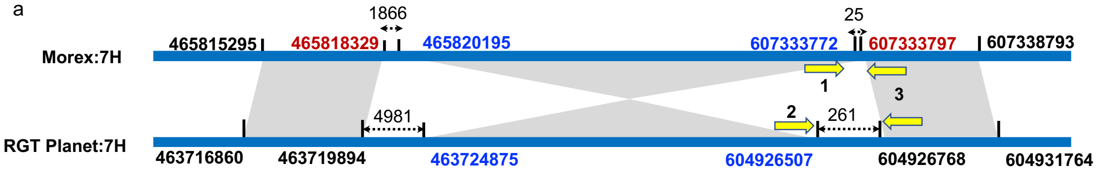

b

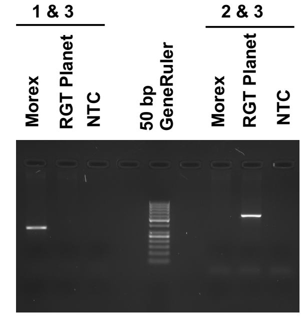

Primer_name	Primer_sequence (5'-3')
7H primer1	ACGACGAGGATGTTTTAACG
7H primer2	CGGACTTGAAGTTCTTTCTTGG
7H primer3	TCTCTCTGTGTGCCAAATGC

c

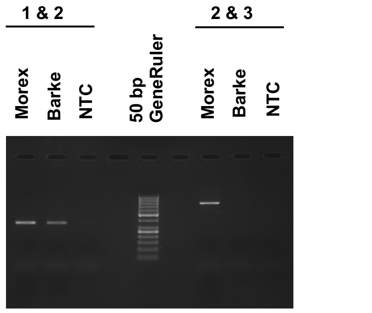

Primer_name	Primer_sequence (5'-3')
2H primer1	ACCAACCAATTTCGTGATGG
2H primer2	TCCTTGTCAACGAGTTCATCC
2H primer3	GTCCCCACTGTGGTACATCC

d

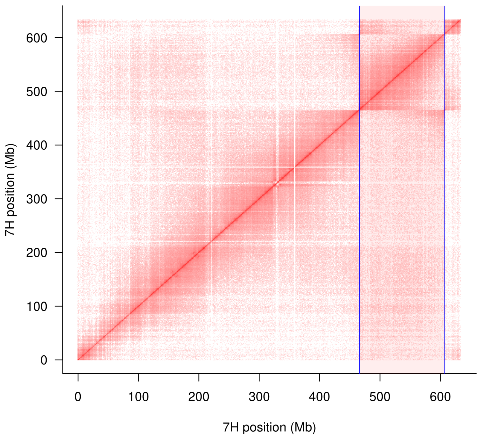

e

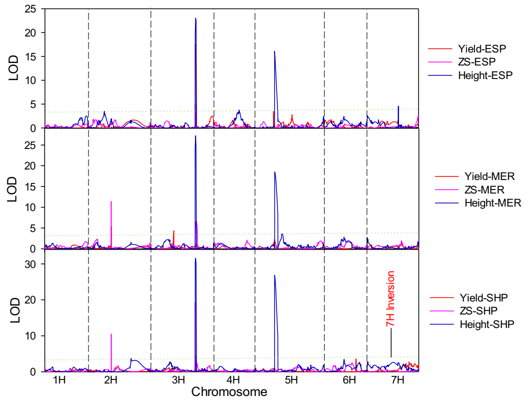

Supplementary Figure 2 | In-depth analysis of two inversions on 2H and 7H. **(a)** Schematic illustration showing precise positions of breakpoints for 7H frequent inversion between Morex and RGT Planet. **(b)** PCR assay for genotyping 7H inversion. The location of three PCR primers are shown in **(a)** with yellow marks (not drawn to scale). **(c)** PCR assay for genotyping the 2H inversion. Primer locations are shown in **Fig. 4c**. **(d)** Hi-C contact probability matrix of RGT Planet computed for chromosome 7H. The intensity of pixels represents the normalized Hi-C links counted between 1 Mb windows on chromosome 7H. The frequent 7H inversion was spotted as a pattern of higher than expected interaction frequency against Morex V2 reference genome, marked by blue lines. **(e)** QTL results for grain yield, plant height and different growth stages from multiple sites in RGT Planet x Hindmarsh population.
