# Supplementary material for: The barley pan-genome reveals the hidden legacy of mutation breeding
Source: Nature. 2020 Nov 25;588(7837):284–9. doi: 10.1038/s41586-020-2947-8 (PMC7759462; doi:10.1038/s41586-020-2947-8)
Supplement: Supplementary file 4 — | PCR-based genotyping of the 7H inversion in the pedigree of RGT Planet. Yellow color denotes carriers of RGT Planet allele. Blue colored cultivars are non-carriers. red color culitvars have unknown status as no fragment was amplified. Cultivars shown in white boxes were not assayed because of unavailability of seeds or DNA. Pedigree data were retrieved from the Barley Pedigree Catalogue (http://genbank.vurv.cz/barley/pedigree/). [file 41586_2020_2947_MOESM4_ESM.pdf]

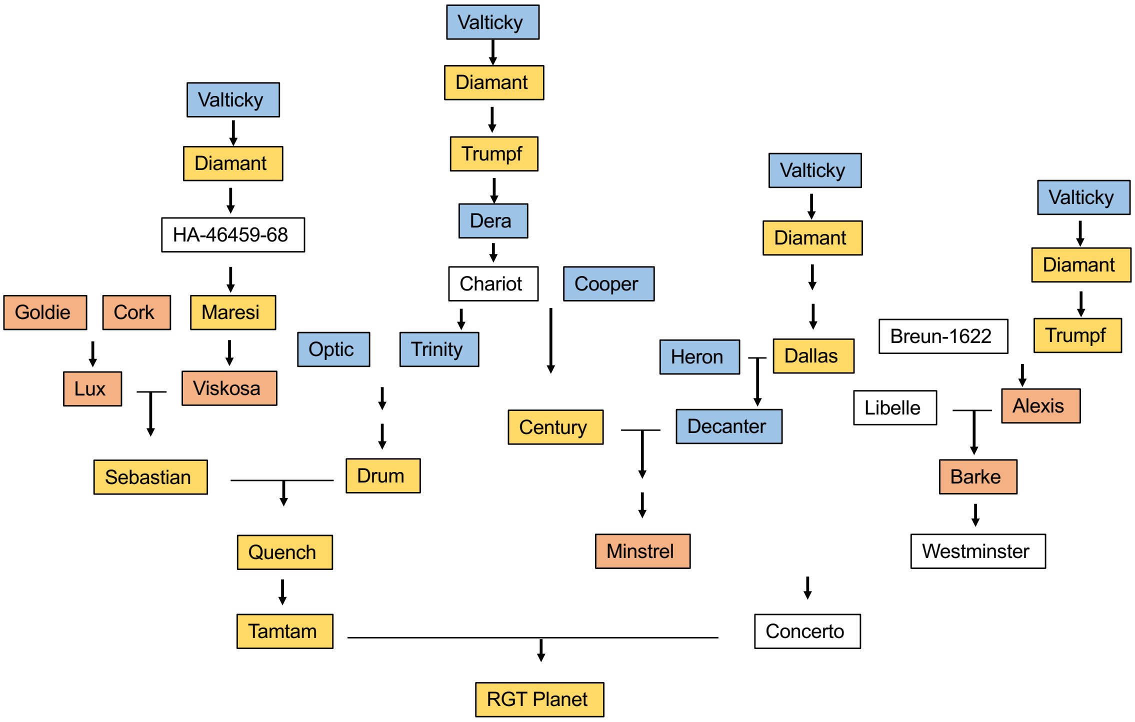

Supplementary Figure 3 | PCR-based genotyping of the 7H inversion in the pedigree of RGT Planet. Yellow color denotes carriers of RGT Planet allele. Blue colored cultivars are non-carriers. red color culitvars have unknown status as no fragment was amplified. Cultivars shown in white boxes were not assayed because of unavailability of seeds or DNA. Pedigree data were retrieved from the Barley Pedigree Catalogue (http://genbank.vurv.cz/barley/pedigree/).
